# Self-assembled supramolecular immunomagnetic nanoparticles through π–π stacking strategy for the enrichment of circulating tumor cells

**DOI:** 10.1093/rb/rbad016

**Published:** 2023-03-09

**Authors:** Yanchao Mao, Yujia Zhang, Yue Yu, Nanhang Zhu, Xiaoxi Zhou, Guohao Li, Qiangying Yi, Yao Wu

**Affiliations:** National Engineering Research Center for Biomaterials, Sichuan University, Chengdu, Sichuan 610064, P. R. China; National Engineering Research Center for Biomaterials, Sichuan University, Chengdu, Sichuan 610064, P. R. China; National Engineering Research Center for Biomaterials, Sichuan University, Chengdu, Sichuan 610064, P. R. China; National Engineering Research Center for Biomaterials, Sichuan University, Chengdu, Sichuan 610064, P. R. China; National Engineering Research Center for Biomaterials, Sichuan University, Chengdu, Sichuan 610064, P. R. China; National Engineering Research Center for Biomaterials, Sichuan University, Chengdu, Sichuan 610064, P. R. China; National Engineering Research Center for Biomaterials, Sichuan University, Chengdu, Sichuan 610064, P. R. China; National Engineering Research Center for Biomaterials, Sichuan University, Chengdu, Sichuan 610064, P. R. China

**Keywords:** circulating tumor cells, magnetic nanoparticles, layer-by-layer self-assembly strategy, π–π, stacking

## Abstract

Owing to their high-specific binding toward targets as well as fast and convenient separation operations, immunomagnetic beads (IMBs) are widely used in the capture and detection of circulating tumor cells (CTCs). To construct the IMBs, surface modifications are generally performed to functionalize the magnetic cores (e.g. Fe_3_O_4_ nanoparticles), and the employed surface modification strategies normally influence the structure and functions of the prepared IMBs in return. Different from the existing work, we proposed the use of supramolecular layer-by-layer (LBL) self-assembly strategy to construct the IMBs. In general, owing to the π–π stacking interactions, the polydopamine, graphene oxide and ‘molecular glue’ γ-oxo-1-pyrenebutyric acid were self-assembled on Fe_3_O_4_ nanoparticles sequentially, thereby accomplishing the integration of different functional components onto magnetic cores to prepare the self-assembled supramolecular immunomagnetic beads (ASIMBs). The ASIMBs showed high sensitivity, specificity and good biocompatibility to the model CTCs and low nonspecific adsorption to the negative cells (∼93% for MCF-7 cells and 17% for Jurkat cells). Meanwhile, ASIMBs possessed a remarkable potential to screen the rare MCF-7 cells out of large amounts of interfering Jurkat cells with the capture efficiency of 75–100% or out of mouse whole blood with the capture efficiency of 20–90%. The captured cells can be further recultured directly without any more treatment, which showed huge applicability of the ASIMBs for *in vitro* detection in clinical practices.

## Graphical Abstract

**Figure:**
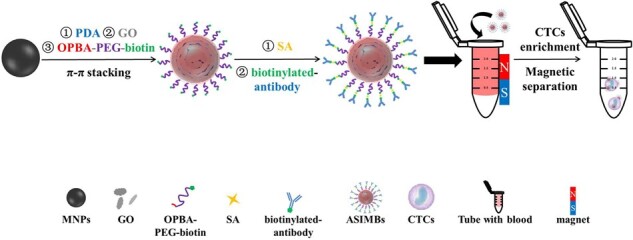

## Introduction

Circulating tumor cells (CTCs), which are shed from the primary tumor tissues or metastatic lesions through an epithelial–mesenchymal transition and then circulate in the peripheral blood until they form a metastatic tumor community in the distant site, were first discovered from a metastatic breast cancer patient by Ashworth in 1869 [1, 2]. As we all know, most cancer-associated deaths and carcinoma recurrence are caused by the metastasis of the primary tumor, which is highly related to the formation and behaviors of the CTCs, and that makes CTCs holding great potential in early detection and diagnosis of cancer, real-time monitoring as well as prognosis of treatment, understanding of drug resistance and recurrence mechanism [3–5]. Although it is significant to detect CTCs, lots of challenges can hardly be ignored. In human’s bloodstream, there are billions of hemocytes involving erythrocytes and leukocytes that will hinder the direct detection of CTCs and may cause significant background signals. On the other hand, CTCs have very low concentrations (several cells in 1 ml of blood), leading to the possible omission of the positive patients [2, 6–8]. Therefore, approaches that hold high sensitivity and specificity to CTCs and can separate CTCs from millions of hemocytes are very much needed.

Different from the traditional invasive detection approaches, liquid biopsy provides novel noninvasive detection methods and has achieved great advances in CTCs’ enrichment [9–10]. These methods can overcome the challenges of CTCs’ separation and detection, and most of them can be divided into two types: (i) based on the unique physical properties of CTCs, such as size [11], deformability [12], density [13] and adhesion [14]; and (ii) the immunoaffinity-based enrichment using antibodies [15], peptides [16] and aptamers [17] as the molecular probes. Although the former has achieved some success, there is no doubt that lots of disadvantages have existed. That is, amounts of large leukocytes have similar physical properties with CTCs, so it is difficult to distinguish them from each other, and these physical approaches always result in low separation efficiency. Immunoaffinity-based enrichment improves the capture efficiency and target accuracy of the materials, and the magnetic nanoparticles (MNPs, e.g. Fe_3_O_4_) are usually combined to prepare immunomagnetic beads (IMBs) for the separation of CTCs due to the fact that these magnetic responsive MNPs can be easily synthesized with low cost and can be combined with antibodies [7, 18, 19], peptides [8, 20] and aptamers [21–23] to improve capture specificity and capture efficiency.

Surface modifications on MNPs are key important factors to determine applicability of the IMBs as well as the cost and reproducibility of manufacturing procedures. Current methods involving cell-engineered strategy [22, 24] and single-layer modification combined with microfluidic devices [25, 26] have been employed to functionalize the MNPs, but they basically require harsh reaction conditions, expensive equipment and raw materials or tedious preparation process that would definitely hinder their wide application. Layer-by-layer (LBL) self-assembly technique that can not only provide high controllable layer coating procedures, but can also combine multiple functionalities in one entity through several coatings with different functions [27, 28], may be one of the optimal candidates to provide a simple and efficient way to overcome the abovementioned disadvantages. For example, Wen *et al*. [18] used LBL methods to construct magnetic nanospheres, where five layers of polyethylenimine as well as nano-γ Fe_2_O_3_ were assembled on the nanospheres to get rapid magnetic response and increased stability. Zhou *et al*. [29] introduced fluorescent quantum dots onto the surface of Fe_3_O_4_ nanoparticles together with other two polyelectrolytes through the electrostatic interaction, thereby enabling an efficient and cell-friendly CTC capture (capture efficiency >90%) as well as visualization of cell recovery.

Despite the success of decorating MNPs through LBL self-assembly using polyelectrolytes, one should notice that the stability of assembled layers driven by electrostatic interactions usually suffer from the changes of environmental pH, ionic strength and temperature and can be easily disassembled by adjusting those factors [30, 31]. In view of this, we proposed that LBL self-assembly based on π–π stacking is more suitable to construct the stable functional layers on MNPs. π–π stacking is a kind of weak interaction widely found in aromatic ring supramolecular systems. It is able to proceed spontaneously, and no complex conditions are generally required. Thus, it is more time-saving, more convenient to operate and lower cost than chemical bonding [32–34]. In theory, self-polymerization of the dopamine molecules under weak alkaline conditions can form stable polydopamine (PDA) coatings on the surface of various materials, due to its superior adhesive nature of amines and catechols along with its film-forming ability [35, 36]. On this basis, PDA can optimize the performance of the matrix material and endow it with hydrophilicity, antifouling performance, biocompatibility, etc., providing an excellent platform for further functional modification of the MNPs. Benefitting from abundant π electron cloud in PDA, π–π stacking interactions can then be utilized to adsorb the substances which containing a large number of aromatic ring structures tightly, typically the graphene oxide (GO) and its derivatives [37, 38]. In return, firmly adsorbed GO can serve as the ‘bridge layer’, to significantly reduce the viscosity of the underlying PDA and also to provide a large number of aromatic rings for next-strep π–π stacking. Although GO has amounts of functional groups such as carboxyl, hydroxyl and epoxy groups, directly combining antibodies with GO is still difficult because of its large hydrophobic plane, and thus several more layers are necessary to introduce the antibodies to the MNPs. γ-oxo-1-pyrenebutyric acid (OPBA), defined as the ‘molecular glue’, can stably link GO through π–π stacking and water-soluble polymer by hydrogen bonding [39, 40]. Considering the existence of the terminal active carboxyl group of OPBA, the self-assembled supramolecular layers (PDA/GO/OPBA) on MNPs can further provide anchor points for antifouling polyethylene glycol (PEG) and the targeting ligands (antibodies).

**Scheme 1.**
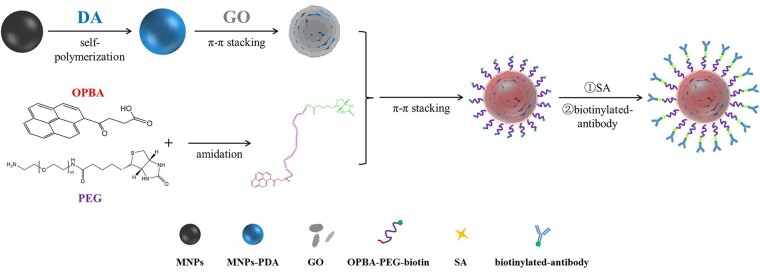
The synthesis procedure of the ASIMBs.

Accordingly, self-assembled supramolecular immunomagnetic beads (ASIMBs) were fabricated through the π–π stacking-driven LBL self-assembly strategy in this work (Scheme 1) and used for highly efficient capture of CTCs. Through the LBL self-assembly process, we successfully constructed ASIMBs which not only had a controllable preparation process that for the most part avoid batch to batch variations but also well-integrated advantages of the building components in a facile way. In large numbers of CTCs’ enrichment, over 90% capture efficiency was obtained by our ASIMBs within 30 min. Besides, 75–93% capture efficiency of rare CTCs from large number of negative cells also proved the high anti-interfering ability of the ASIMBs, further revealing the proposed detection platform had high capture efficiency, good specificity and high sensitivity. Meanwhile, the CTCs captured by ASIMBs can be directly transferred to Petri dishes for culture, and the proliferation and morphology of cells are slightly influenced by the ASIMBs, which showed its promising applicability in biological samples.

## Experimental section

### Materials and reagents

Iron (III) chloride hexahydrate (FeCl_3_·6H_2_O), sodium acetate, dopamine (DA) and N,N-iisopropylethylamine (DIPEA) were purchased from Sigma Aldrich (St Louis, MO, USA). OPBA, biotinylated polyethylene glycol amine (NH_2_-PEG-biotin, Mw 2000) and 1-ethyl-3-(3′-dimethylaminopropyl) carbodiimide hydrochloride (EDCI) were purchased from Aladdin (Shanghai, China). Sodium citrate was purchased from Kelong Chemicals Co., Ltd. (Chengdu, China). Ethylene glycol was purchased from Zhiyuan Chemical Reagent Co., Ltd. (Tianjin, China). GO (per size ∼500 nm) was purchased from Nanjing Jicang Nano Technology Co., Ltd. (Nanjing, China). 1-hydroxybenzotriazole (HOBt) was purchased from GL Biochemical (Shanghai) Co. Ltd. (Shanghai, China). Anhydrous N,N-dimethylformamide (anhydrous DMF) was purchased from Titan Chemical Co., Ltd. (Shanghai, China). Streptavidin (SA) and FITC-conjugated streptavidin (SA-FITC) were purchased from Solarbio (Beijing, China). Biotin-anti-human-epithelial-cell-adhesion-molecule (biotinylated anti-EpCAM) antibody was purchased from Cell Signaling (Danvers, USA). Goat anti-mouse IgG-FITC antibody was purchased from Biolegend (San Diego, USA). Dulbecco’s modified eagle medium (DMEM) and Roswell Park Memorial Institute-1640 medium (RPMI-1640) were purchased from Thermo Fisher (Shanghai, China).

### Preparation of the MNPs@PDA@GO nanoparticles

First, the MNPs were synthesized using a hydrothermal method [23]. Briefly, 2.04 g FeCl_3_·6H_2_O, 3.6 g sodium acetate and 0.6 g sodium citrate were mixed with 60 ml ethylene glycol in a teflon-sealed autoclave for ∼1 h to form a homogeneous mixture. Afterwards, the mixture was reacted for 12 h at 100°C along with one more hour’s temperature maintenance. After cooling down to room temperature, the black solid products were washed with ethanol and deionized water for three times along with a magnetic separation procedure, respectively. The obtained MNPs were stored in the deionized water at 4°C before use.

Then, two modification steps were carried out to functionalize the MNPs [37, 38]. First, to generate the PDA layer, 10 mg DA was dissolved with 10 ml Tris buffer (10 mM, pH = 8.2) and sonicated for ∼3 min. After washing several times with Tris buffer, 2.5 ml MNPs (∼100 mg) were added into the DA solution and the final volume of the mixture was set to 30 ml. The reaction was carried out under room temperature with mechanical mixing (450 rpm) for 6 h, and the products were washed with deionized water for three times along with a magnetic separation procedure. Later, to deposit the GO layer, 1.5 ml MNPs@PDA (∼12 mg) were diluted with deionized water to 1 mg·ml^−1^ and 1.2 ml GO aqueous solution (0.6 mg) were added along with ultrasonic for 1 min. After that, the solution was vigorously stirred for 6 h and the products were washed with deionized water for three times along with a magnetic separation procedure. The obtained MNP@PDA@GO was stored in the deionized water at 4°C before use.

### Construction of the antibody functionalized superparamagnetic nanoparticles

Strategically, the component OPBA-PEG-biotin will be introduced onto the surface of MNP@PDA@GO through the ‘π-π stacking’ between OPBA and GO [40]. For this purpose, OPBA was first linked to the NH_2_-PEG-biotin through the amidation reaction between amino and carboxyl. Briefly, 30 mg OPBA, 237 mg NH_2_-PEG-biotin, 27.3 mg EDCI and 19.2 mg HOBt were added into an eggplant-shaped flask. With the nitrogen protection, an anhydrous DMF solution (15 ml) containing 25.05 μl DIPEA was added into the flask drop by drop for ∼30 min. Then, the whole system was left to react at room temperature with magnetic stirring for 48 h, accompanied by a 72 h dialysis procedure using deionized water to remove the excessive reactant. The obtained product (OPBA-PEG-biotin) was then reacted with MNP@PDA@GO to accomplish the ‘molecular glue’-mediated assembly. In brief, MNP@PDA@GO (12 ml, 6 mg) and OPBA-PEG-biotin (8 ml, 40 mg) were mixed together and shaken gently for 6 h. After the reaction, the products were washed with PBS for three times along with a magnetic separation procedure to produce the MNP@PDA@GO@OPBA-PEG-biotin.

Moreover, 1 ml MNP@PDA@GO@OPBA-PEG-biotin suspension and 16 μl 5 mg·ml^−1^ SA were mixed and left to react at room temperature for 1 h with gentle shaking. The products were washed with PBS for twice along with a magnetic separation procedure. Last, 1 ml MNP@PDA@GO@OPBA-PEG-SA suspension and 12 μl 0.5 mg·ml^−1^ biotinylated anti-EpCAM antibody were mixed and left to react at 4°C for 1.5 h with gentle shaking. The final products, named as the ASIMBs, were washed with PBS for twice along with a magnetic separation procedure and stored in the PBS at 4°C [41].

For calculating the amounts of antibodies combined with MNPs, the fluorescence intensity at 520 nm of 1, 2, 3, 4, 5 and 6 μg·ml^−1^ goat anti-mouse IgG-FITC antibody was tested in the excitation wavelength of 488 nm, and the standard fluorescence emission curve of goat anti-mouse IgG-FITC antibody was acquired. Then, the fluorescence intensity at 520 nm of 1 ml ASIMBs (∼1 mg) was tested in the excitation wavelength of 488 nm, and the concentration of antibodies combined with 1 ml ASIMBs could be calculated with the equation of standard fluorescence emission curve.

Besides, we confirmed the combination of OPBA-PEG-biotin and biotinylated anti-EpCAM antibody on the MNPs using the confocal laser scanning microscopy (CLSM). On one hand, 1 mg MNP@PDA@GO@OPBA-PEG-biotin and 1 mg MNP@PDA@GO were mixed with 80 μg SA-FITC, respectively, and left to react at room temperature for 1 h with gentle shaking. The products were washed with PBS for three times along with a magnetic separation procedure. Then, ∼5 μg·ml^−1^ final products were observed under the CLSM. On the other hand, 1 mg MNP@PDA@GO@OPBA-PEG-SA-antibody and MNP@PDA@GO@OPBA-PEG-SA were mixed with 12 μg goat anti-mouse IgG-FITC antibody and left to react at 37°C for 0.5 h with gentle shaking. The products were washed with PBS for three times along with a magnetic separation procedure. Then, ∼5 μg·ml^−1^ final products were observed under the CLSM.

### Cell culture

All the culture medium including DMEM and RPMI-1640 were added 10% FBS and 1% penicillin-streptomycin before use. The DMEM medium was used for MCF-7, HepG2 cells, while the RPMI-1640 medium was used for Jurkat cells. For MCF-7 and HepG2 cells, when the cells colonized over 90% area of the Petri dishes, cells were trypsin-digested to perform the passaging procedure. For Jurkat cells, when the cells colonized over 90% area of the Petri dishes, a 250 g centrifugal operation was conducted to perform the passaging procedure. All the cells were cultured in a humidified atmosphere of 5% CO_2_ at 37°C.

### Capture efficiency and the specificity of the ASIMBs

The concentration of ASIMBs and the incubation time were selected to optimize the capture efficiency of ASIMBs. For the optimal concentration of ASIMBs, five different concentrations (15, 30, 60, 90 and 120 μg·ml^−1^) were set, and ∼1 × 10^6^ MCF-7 cells were added into each group. After incubating at 4°C for 30 min with gentle shaking, the suspension was magnetic separated for 2 min, and cells in the supernatant were added into the cell counting chamber (∼20 μl of 1 ml supernatant). Then, the numbers of cells in the supernatant were counted by the cell counter (Counterstar BioTech, China) with the set program (six areas of each group were randomly selected to obtain the average cell numbers). For the incubation time, five different times (5, 10, 20, 30 and 60 min) were set. For a certain time, 60 μg·ml^−1^ of ASIMBs was incubated with 1 × 10^6^ MCF-7 cells at 4°C with gentle shaking. The suspension was magnetic separated for 2 min, and the numbers of cells in the supernatant were counted as mentioned above.

To verify the capture specificity of the ASIMBs, a two steps’ experiment involving different kinds of materials and different types of cells was designed and performed [42, 43]. On one hand, 60 μg·ml^−1^ MNP@PDA@GO, MNP@PDA@GO@OPBA-PEG-biotin and ASIMBs were incubated with 1 × 10^6^ MCF-7 cells. After incubating at 4°C for 30 min with gentle shaking, the suspension was magnetic separated for 2 min, and the numbers of cells in the supernatant were counted as mentioned above. On the other hand, 60 μg·ml^−1^ of ASIMBs were incubated with 1 × 10^6^ MCF-7 cells, HepG2 cells and Jurkat cells. After incubating at 4°C for 30 min with gentle shaking, the suspension was magnetic separated for 2 min, and the numbers of cells in the supernatant were counted as mentioned above.

The capture efficiency of each experiment was calculated by: where *A*_0_ is the numbers of cells in the suspension before capture, and *A*_1_ is the numbers of cells in the supernatant after capture.


(1) $$\mathrm{Capture}\;\mathrm{efficiency}\;\left(\%\right)=\frac{A_{0}-A_{1}}{A_{0}}\times 100\%,$$

### **Capture of CTCs from** the mimicked biological samples

To mimic the real situation of blood environment and examine the capture limit of ASIMBs, we chose MCF-7 cells as the model cells and Jurkat cells as the interfering cells or mouse whole blood as the mimicked environment to perform the experiment. Firstly, MCF-7 cells were dyed with Hochest. Then, 3, 5, 10, 20, 50, 100, 150 and 200 prestained MCF-7 cells were mixed with 1 × 10^6^ Jurkat cells, and 3, 5, 10, 20, 50 and 100 prestained MCF-7 cells were mixed with 1 ml mouse whole blood. The mixed cells were incubated with 60 μg·ml^−1^ ASIMBs at 4°C for 30 min with gentle shaking. Finally, the suspension was magnetic separated for 2 min, and the MCF-7 cells in the precipitate were observed and counted by CLSM.

### Characterizations

Scanning electron microscope (SEM, Hitachi S-4800, Japan) was applied to observe the morphology of the prepared samples including materials as well as cells incubated with materials and acquire the energy dispersive spectroscopy of cells incubated with materials. Transmission electron microscope (TEM, TALOS F200S G2, Czech) was used to obtain the detail morphology of each modification layers of ASIMBs. The nanoparticles’ size distribution and Zeta potential were measured by dynamic light scattering (DLS, Zteasizer Nano ZS90, Malvern Company). The confocal images were obtained using confocal laser scanning microscopy (CLSM, Leica TCP SP5, Germany). UV-vis spectrophotometer (Hitachi, U-2910, Japan) was applied to evaluate materials’ magnetic recovery efficiency. Hysteresis loop characterization of magnetic materials was conducted by vibrating sample magnetometer (VSM, LakeShore7404, Quantum Design PPMS DynaCool, USA). The fluorescence spectrum was obtained using molecular Fluorescence Spectrometer (FL, Horiba, Fluorolog-3, France). To acquire the capture efficiency of each experiment, the cell-counter (Counterstar BioTech, China) was used to read out the cells’ number before and after the incubation, and each group was counted six times to avoid accidental error.

## Results and discussion

### Synthesis and characterizations of the ASIMBs

**Figure 1.**
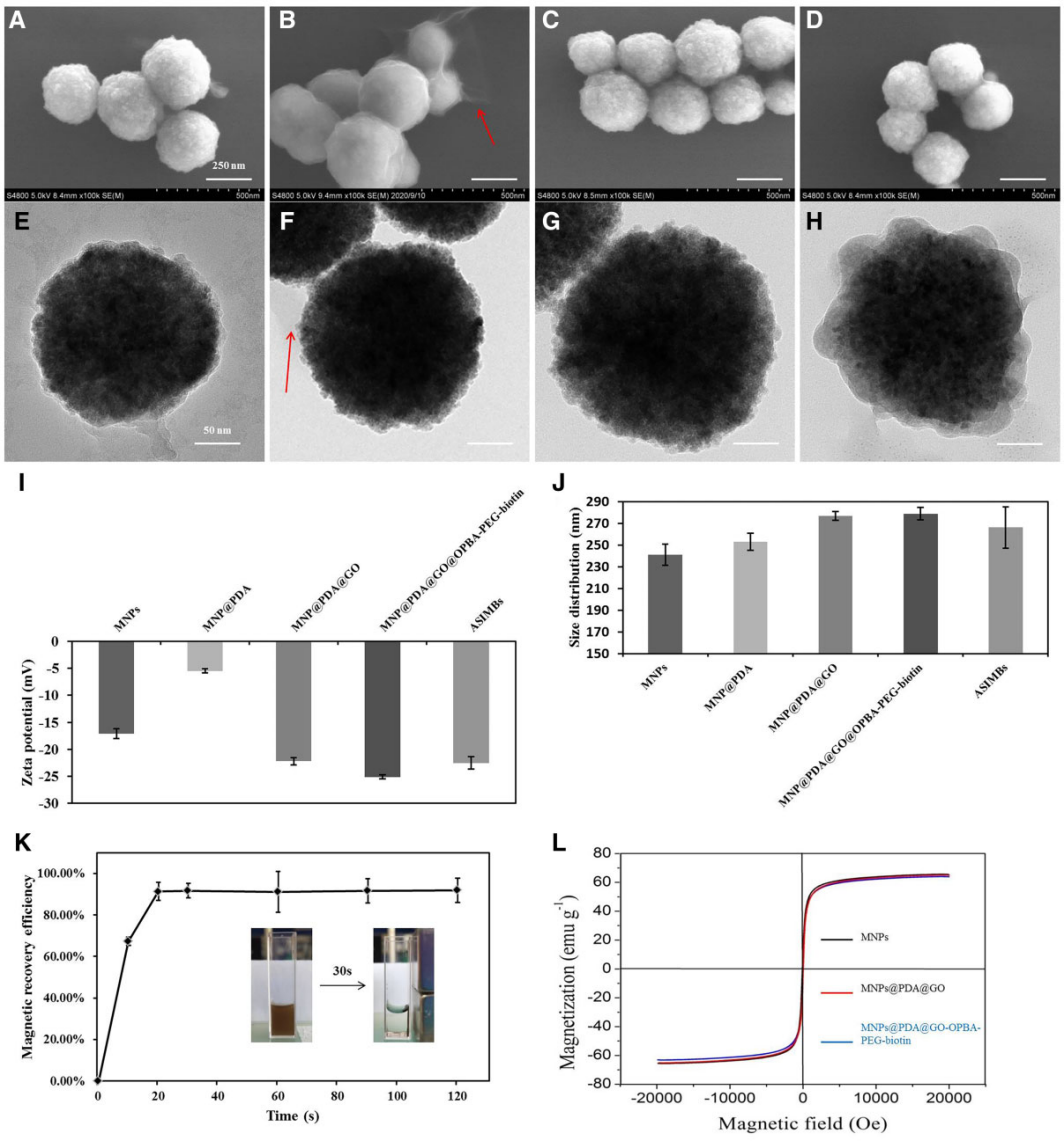
Characterization of the nanoparticles. SEM images of the (**A**) MNPs@PDA, (**B**) MNPs@PDA@GO, (**C**) MNPs@PDA@GO@OPBA-PEG-biotin, (**D**) ASIMBs; TEM images of the (**E**) MNPs@PDA, (**F**) MNPs@PDA@GO, (**G**) MNPs@PDA@GO@OPBA-PEG-biotin, (**H**) ASIMBs, and the arrows showed the ‘chiffon-liked’ GO around the MNPs. (**I**) Size distribution and (**J**) Zeta potential changes of nanoparticles during the fabrication process. The measurement was performed in water with the pH = 7, and error bars represent the standard deviations (*n* = 3). (**K**) Magnetic recovery efficiency of ASIMBs at different time with a commercial magnetic scaffold, the inset graph showed the magnetic separation process of ASIMBs within 30 s. Error bars represent the standard deviations (*n* = 5). (**L**) Magnetic hysteresis loops of the MNPs, MNPs@PDA@GO and MNPs@PDA@GO@OPBA-PEG-biotin measured at room temperature.

MNPs were synthesized using a hydrothermal method with a little modification to obtain stable and well-dispersed Fe_3_O_4_ nanoparticles, and then three functional layers including PDA, GO and OPBA-PEG-biotin were introduced to the MNPs. PDA was self-polymerized on the surface of MNPs, and after that GO was combined with PDA and OPBA-PEG-biotin through π–π stacking. Finally, antibodies were introduced onto MNPs through the reaction between SA and biotin. Corresponding changes in morphology, size distribution and Zeta potential were carefully recorded (Fig. 1A–J). As the DA polymerized on the surface of MNPs, the obtained nanoparticles (MNP@PDA) became rougher with a ∼3 nm polymer layer displayed under microscope observations (Fig. 1A and E); their size distribution changed from 240.9 to 252.9 nm and Zeta potential increased from −17.10 mV to −5.47 mV due to generation of the PDA layer on the surface (Fig. 1I and J). Besides, we have successfully introduced PDA onto MNPs surface in 2D plane (Supplementary Fig. S3), which confirms the feasibility of DA’s self-polymerization on the surface of MNPs. Subsequently, after GO deposition, obvious ‘chiffon-liked’ films were attached to the nanoparticles (Fig. 1B and F, where the red arrows showed the ‘chiffon-liked’ GO around the MNPs), and the obtained MNP@PDA@GO showed an increased average diameter (276.7 nm) and Zeta potential decreased to about −20 mV because of abundant carboxyl and hydroxyl on GO (Fig. 1I and J). Theoretically, the GO layers on magnetic cores would enlarge specific surface area for cells adhesion and provide multiple modification way to introduce functional components.

**Figure 2.**
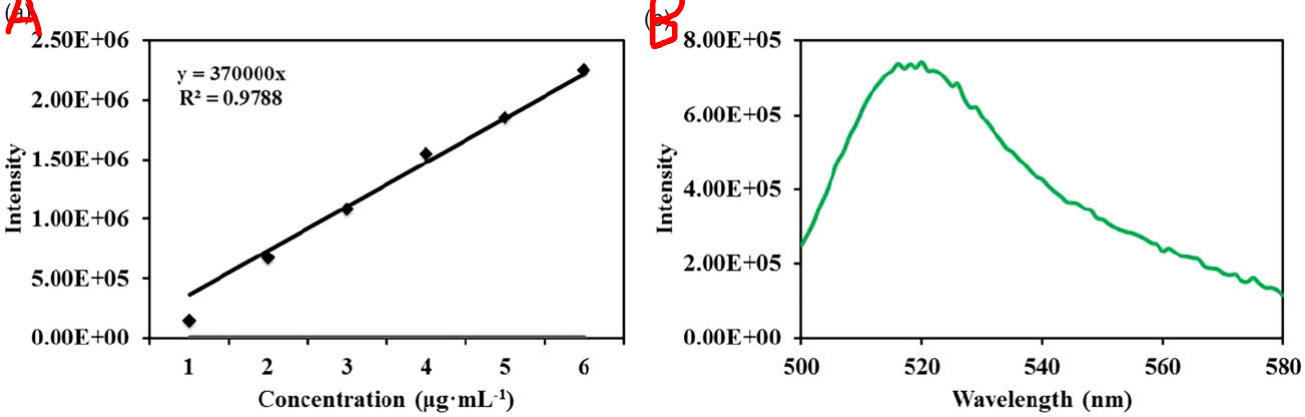
(**A**) The standard emission curve of anti-mouse IgG-FITC in PBS at room temperature. (**B**) FL curve of 1.0 mg ASIMBs after incubated with anti-mouse IgG-FITC in PBS at room temperature.

OPBA was defined as the ‘molecular glue’ in our work, to glue the GO-functionalized magnetic cores and antibody components together. On one hand, OPBA could efficiently and stably combine with GO through ‘π-π stacking’. On the other hand, the terminal of OPBA had active carboxyl group which could easily react with amino, hydroxyl and other groups. With the strong binding ability of OPBA, the magnetic cores and the OPBA-PEG-biotin were tightly bonded. Later, combination of the obtained MNP@PDA@GO@OPBA-PEG-biotin with SA and biotinylated anti-EpCAM antibody accomplished the construction of the ASIMBs system. In order to minimize possible aggregation caused by the interaction of SA and biotin, MNP@PDA@GO@OPBA-PEG-biotin suspension was added into the SA solution dropwise over a period of 30 min, with the SA/biotin molar ratio of ∼1:10. Then, magnetic isolation was conducted to purify the product. Similar manipulation was performed for further introduction of biotinylated anti-EpCAM antibody. Amount of the conjugated antibody on 1.0 mg ASIMBs was detected to be 2.01 µg, according to the fluorescence spectroscopy analyses with corresponding FITC-labeled anti-mouse IgG (Fig. 2). ^1^H NMR was applied to confirm the product of OPBA-PEG-Biotin. As shown in Supplementary Fig. S2, the chemical shifts at δ 3.44 (ppm) confirm the successful conjugation of the NH2-PEG-biotin and OPBA (red arrows), and the chemical shifts around δ 3.70 (ppm) represent the 4H in PEG (green arrows). SEM and TEM measurements evidenced the clear morphology change after antibody introduction, where slightly thicker low-contrast layers accumulated on the MNP@PDA@GO@OPBA-PEG-biotin were found (Fig. 1D and H), though no huge changes on particles’ Zeta potential were detected (Fig. 1I and J). It should be noted, the particles’ average size decreased slightly after antibody conjugation (Fig. 1I), though corresponding size distribution showed no obvious change. Reasonable explanation should be attributed to the introduction of antibodies, which possessed better water-binding capability to disperse the prepared particles in PBS when performing the DLS characterization. In addition, the prepared ASIMBs showed good magnetic response upon exposure to external magnetic field. As shown in Fig. 1K, the well-dispersed ASIMBs can be separated rapidly within 30 s, and the recovery efficiency reached ∼92% when the magnetic exposure time was 30 s. This result was highly supported by the VSM test, where the MNP@PDA@GO@OPBA-PEG-biotin maintained its magnetic well and possessed a relatively high-magnetic saturation value (61.4 emu/g) even after several modification steps. These results endowed the good magnetic responsive property to the ASIMBs, which could simplify the reprocessing procedure and separate the captured cells rapidly and efficiently.

**Figure 3.**
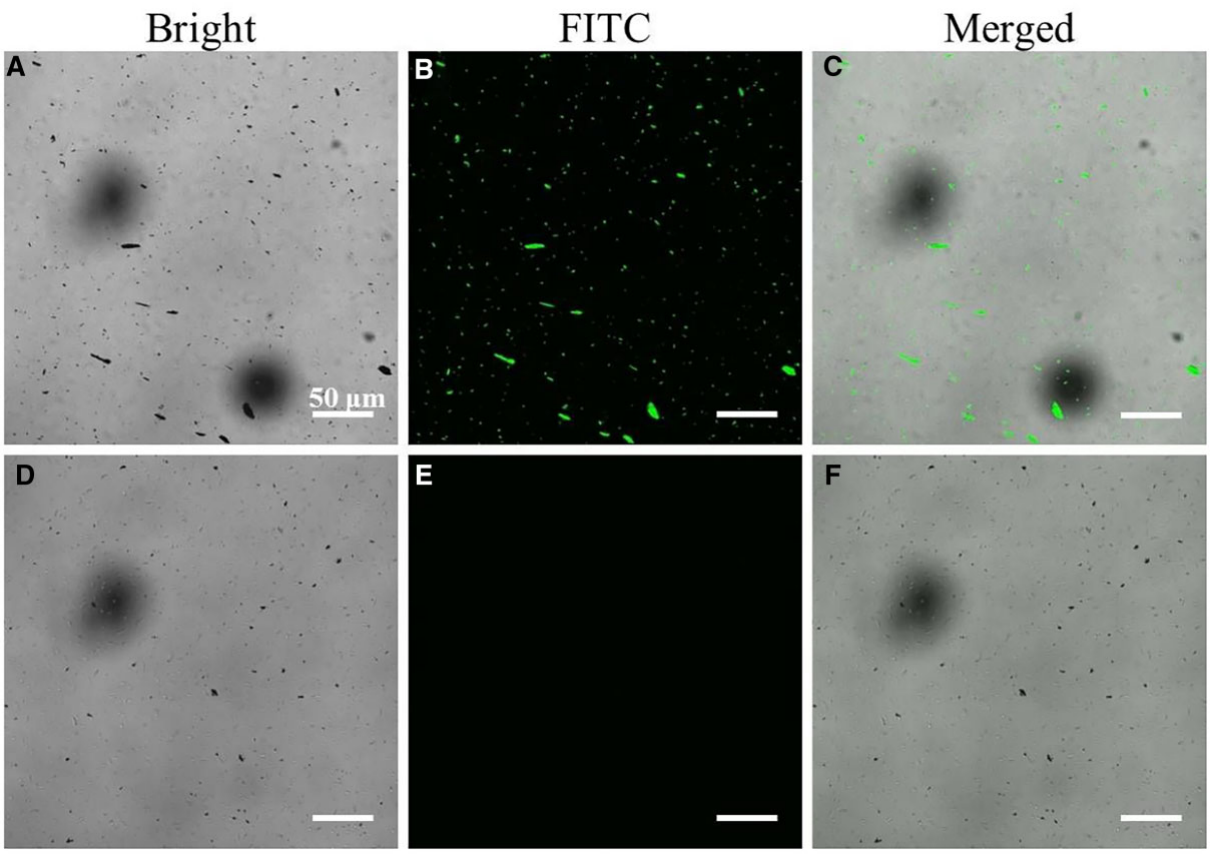
(**A**–**C**) CLSM images of MNPs@PDA@GO@OPBA-PEG-biotin + SA-FITC. (**D**–**F**) CLSM images of MNPs@PDA@GO + SA-FITC.

**Figure 4.**
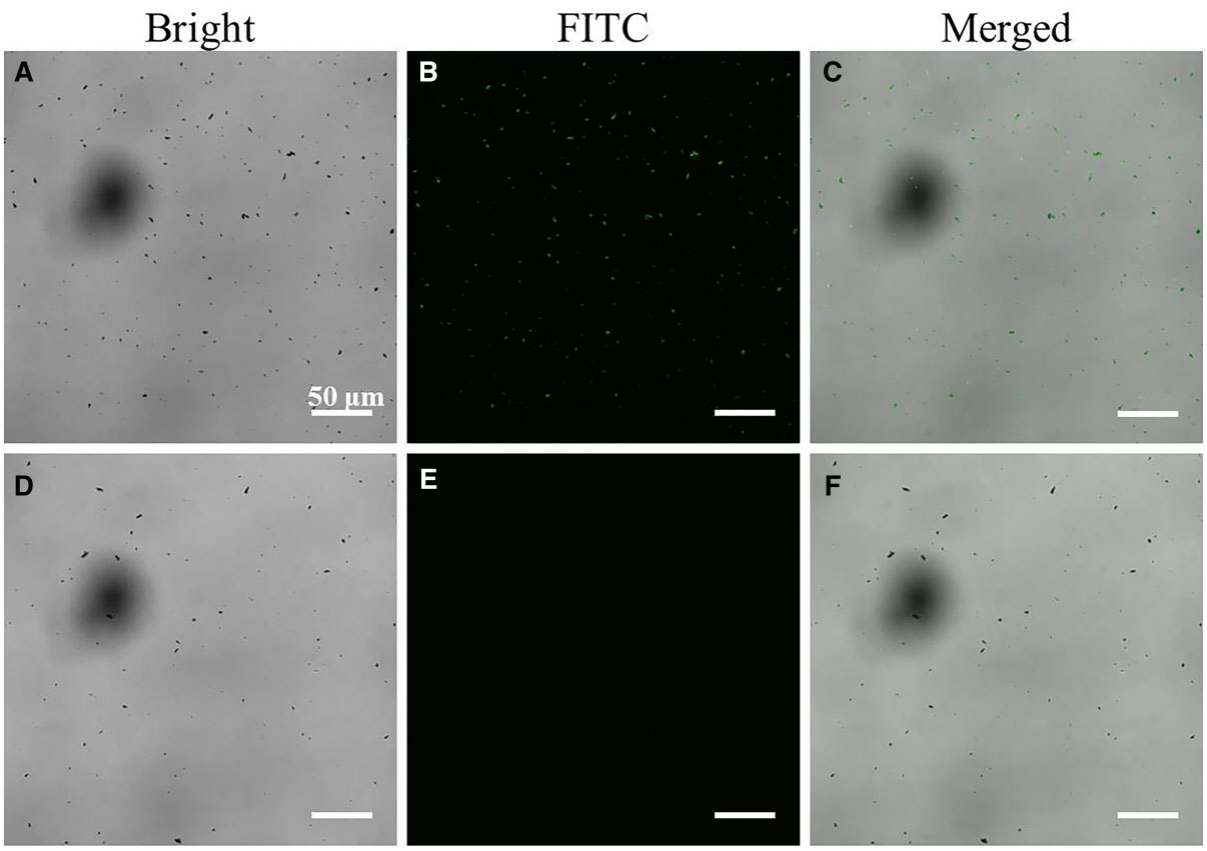
(**A**–**C**) CLSM images of MNPs@PDA@GO@OPBA-PEG-biotin + biotinylated anti-EpCAM antibody + goat anti-mouse IgG-FITC antibody. (**D**–**F**) CLSM images of MNPs@PDA@GO@OPBA-PEG-biotin + goat anti-mouse IgG-FITC antibody.

To further verify the combination of the OPBA-PEG-biotin and the biotinylated anti-EpCAM antibody, we adapted fluorescent small molecules FITC to make the results visual. Firstly, SA-FITC was reacted with the biotin functional group as the same way of the SA. Obvious fluorescence could be seen from the MNP@PDA@GO@OPBA-PEG-biotin + SA-FITC group, while nearly no fluorescence existed around the MNP@PDA@GO + SA-FITC group (Fig. 3). The results confirmed the successful introduction of the OPBA-PEG-biotin as well as the reaction between the biotin and SA. In view of this, biotinylated anti-EpCAM antibody can therefore be introduced to prepare fully functionalized ASIMBs for the ultimate object of the capture of CTCs, and the successful conjugation and bioactivity of the antibody component were detected by CLSM, by whom the interaction of biotinylated anti-EpCAM antibody and its corresponding fluorescent secondary antibody (goat anti-mouse IgG-FITC antibody) were recorded. As shown in Fig. 4A–C, the MNP@PDA@GO@OPBA-PEG-biotin with biotinylated anti-EpCAM antibody emitted strong green fluorescent signals upon incubating with goat anti-mouse IgG-FITC antibody. On the contrast, without the biotinylated anti-EpCAM antibody, no obvious fluorescent signal can be detected (Fig. 4D–F).

### Optimum incubation conditions of the ASIMBs for MCF-7 cells

**Figure 5.**
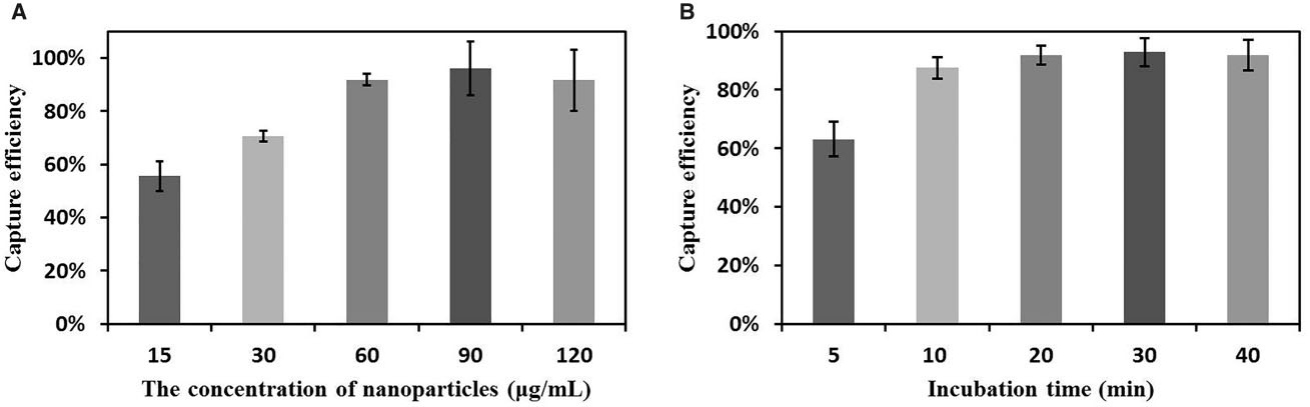
(**A**) Influence of the concentration of ASIMBs to the capture efficiency of ASIMBs. The incubation time was set to 30 min and 1 × 10^6^ MCF-7 cells were dispersed in PBS for capture. (**B**) Influence of the incubation time to the capture efficiency of ASIMBs. The concentration of ASIMBs was set to 60 μg/ml and 1 × 10^6^ MCF-7 cells were dispersed in PBS for capture. Error bars represent the standard deviations (*n* = 5).

Before applying our materials to capture cells in different samples, we explored the optimum capture conditions of ASIMBs involving the concentration of ASIMBs and the incubation time. First, studies were carried out in MCF-7 system to optimize the ASIMBs concentration for the capture of CTCs. As shown in Fig. 5A, the capture efficiency increased rapidly as the concentration of nanoparticles rising from 15 to 60 μg/ml, and few obvious changes could be seen as the concentration continuing to increase. The maximum capture efficiency was found to be 96.10% when 90 μg/ml ASIMBs was applied. Considering that excessive nanoparticles were too costly and might exist potential biological toxicity, we eventually chose 60 μg/ml as the optimum concentration for further studies. Next, five different times were set to explore the optimum incubation time (Fig. 5B). The capture efficiency reached to ∼92.90% within 30 min, and emerged no obvious increase as the time extending continually. So, 30 min was chosen as the optimum incubation time for the further CTCs capture experiments.

### Capture capability and specificity of ASIMBs

**Figure 6.**
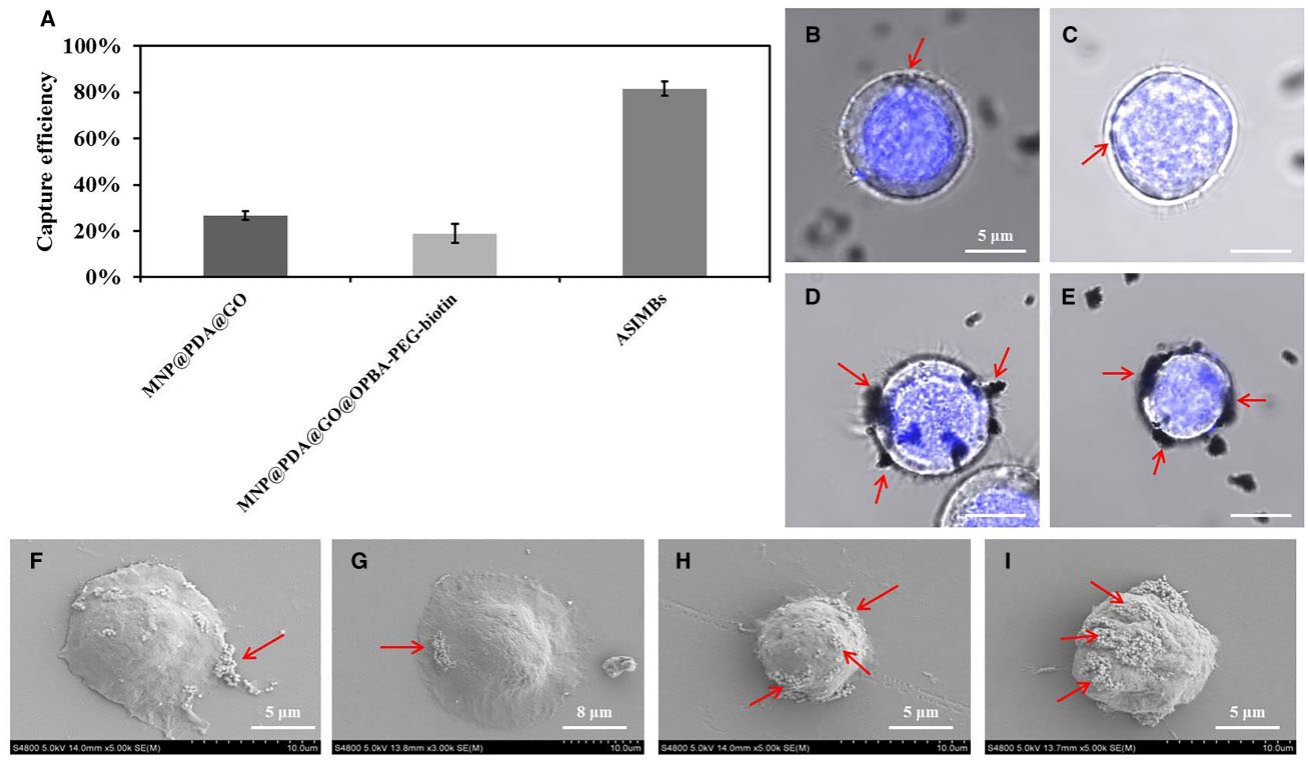
(**A**) Capture efficiency of MNPs@PDA@GO, MNPs@PDA@GO@OPBA-PEG-biotin and ASIMBs for 1 × 10^6^ MCF-7 cells in PBS for 30 min, and the concentration of three groups was 60 μg/ml. Error bars represent the standard deviations (*n* = 5); CLSM images of MCF-7 cells incubated with (**B**) MNPs@PDA@GO, (**C**) MNPs@PDA@GO@OPBA-PEG-biotin, (**D**) 60 μg/ml ASIMBs and (**E**) 120 μg/ml ASIMBs. The arrows showed the nanoparticles that were around the MCF-7 cells; SEM images of MCF-7 cells incubated with (**F**) MNPs@PDA@GO, (**G**) MNPs@PDA@GO@OPBA-PEG-biotin, (**H**) 60 μg/ml ASIMBs and (**I**) 120 μg/ml ASIMBs. The arrows showed the nanoparticles that were combined with the MCF-7 cells.

Above experiments had demonstrated that the ASIMBs were capable of capturing the cells efficiently with high capture efficiency over 90% for large numbers of MCF-7 cells (∼1 × 10^6^ cells) within 30 min, with the help of conjugated antibodies to recognize the target cells and the magnetic cores to enable fast magnetic separation. Besides, considering the rarity of CTCs in the human’s peripheral blood and possible interference from surrounding abundant amount of hemocytes and proteins, it is necessary to guarantee the materials’ specificity for enriching the CTCs [2–4]. Strategically, the biotinylated anti-EpCAM antibody was employed to provide high-specific binding toward the epithelial cell adhesion molecules that were overexpressed on tumor cells of the epithelial type, like the MCF-7 cells. To verify this, nanomaterials with/without the antibodies (MNP@PDA@GO, MNP@PDA@GO@OPBA-PEG-biotin and ASIMBs) were incubated with 1 × 10^6^ MCF-7 cells, and the capture efficiency was detected. As shown in Fig. 6A, without antibodies, the MNP@PDA@GO group captured ∼39% cells. Such model CTCs capture result may mainly attribute to the nonspecific adsorption, and this side effect was obviously decreased (capture efficiency of MNP@PDA@GO@OPBA-PEG-biotin was 20.65%) when the antifouling component PEG was introduced on the nanoparticles [44]. On the contrast, the fully functionalized ASIMBs showed the highest capture efficiency of 90.59%. Moreover, through the CLSM and the SEM photographs, the high-specific binding of the ASIMBs was further evidenced. It was clear, nonspecific adsorption led to the existence of small amounts of nanoparticles around the model CTCs (Fig. 6B, C, F and G), while the antibody-mediated CTCs recognition resulted in more evenly distributed nanoparticles on cells. Meanwhile, increase of the added ASIMBs numbers showed a dosage-dependent nanoparticle binding on the model CTCs (Fig. 6D, E, H and I). All these results revealed that our materials with antibodies conjugation had higher affinity to the target cells, and would take significant role in the capture of CTCs.

**Figure 7.**
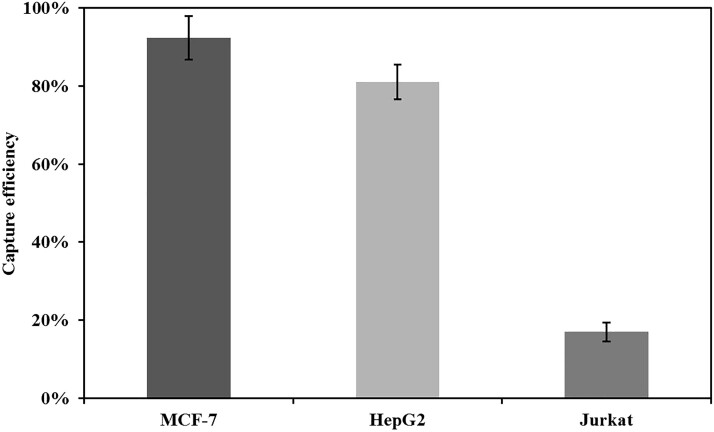
Capture efficiency of ASIMBs (60 μg/ml) for three different cells (1 × 10^6^) within 30 min in PBS. Error bars represent the standard deviations (*n* = 5).

Moreover, the general applicability of our ASIMBs was investigated. In brief, three different cells, the MCF-7 cells, HepG2 cells and Jurkat cells with varied EpCAM expression level, were chosen to validate the ASIMBs’ CTCs capture specificity. As shown in Fig. 7, only MCF-7 cells and HepG2 cells that overexpressed epithelial cell adhesion molecules could be captured more than 80%, and the capture efficiency of the MCF-7 cells was over 90%, which might be due to higher expression of epithelial cell adhesion molecules on the MCF-7 cells than HepG2 cells. On the other hand, Jurkat cells, a kind of acute T-cell leukemia cells that expressed nearly no epithelial cell adhesion molecules, obtained only 17% capture efficiency, which was much lower than the positive cells. This experiment again confirmed our materials’ specificity to the tumor cells of the epithelial type, especially for the MCF-7 cells, and was important for the further clinical application.

Further, one should notice that even though better capture specificity of the ASIMBs toward epithelial CTCs was achieved when compared with current representative research works (Supplementary Table S1), the capability of the ASIMBs to antifouling of abundant surrounding interfering cells still needs to be strengthened, thereby satisfying the applications in clinical trials, where both high-specificity and high-purity capture of CTCs are required.

### Capture of CTCs **from** the mimicked biological samples

**Figure 8.**
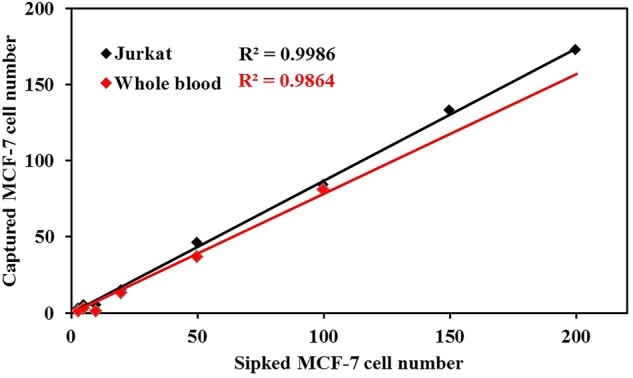
Captured MCF-7 cell number with 60 μg·ml^−1^ ASIMBs, where 3, 5, 10, 20, 50, 100, 150 and 200 MCF-7 cells were spiked into 1 × 10^6^ Jurkat cells in PBS and 3, 5, 10, 20, 50 and 100 MCF-7 cells were spiked into mouse whole blood.

To test the anti-interference ability and define the detection limit of ASIMBs, small amounts (3–200) of prestained MCF-7 cells were spiked into 1 ml of either the interfering cells (1 × 10^6^ Jurkat cells in PBS) or the mouse whole blood. As shown in Fig. 8A, ASIMBs’ capture capability for MCF-7 cells was little interfered by the amounts of negative cells even when the amount of MCF-7 cells was <10, and the capture efficiency was 75–100% for MCF-7 cells in 1 × 10^6^ of Jurkat cells and 20–90% for MCF-7 cells in 1 ml of whole mouse blood. Meanwhile, the number of captured MCF-7 cells showed a good linear relationship to the spiked MCF-7 cell numbers between 20 and 500 cells (*R*^2^ = 0.998 or 0.986), which revealed ASIMBs’ detection accuracy for CTCs and possess huge capability for CTCs’ quantity detection.

**Figure 9.**
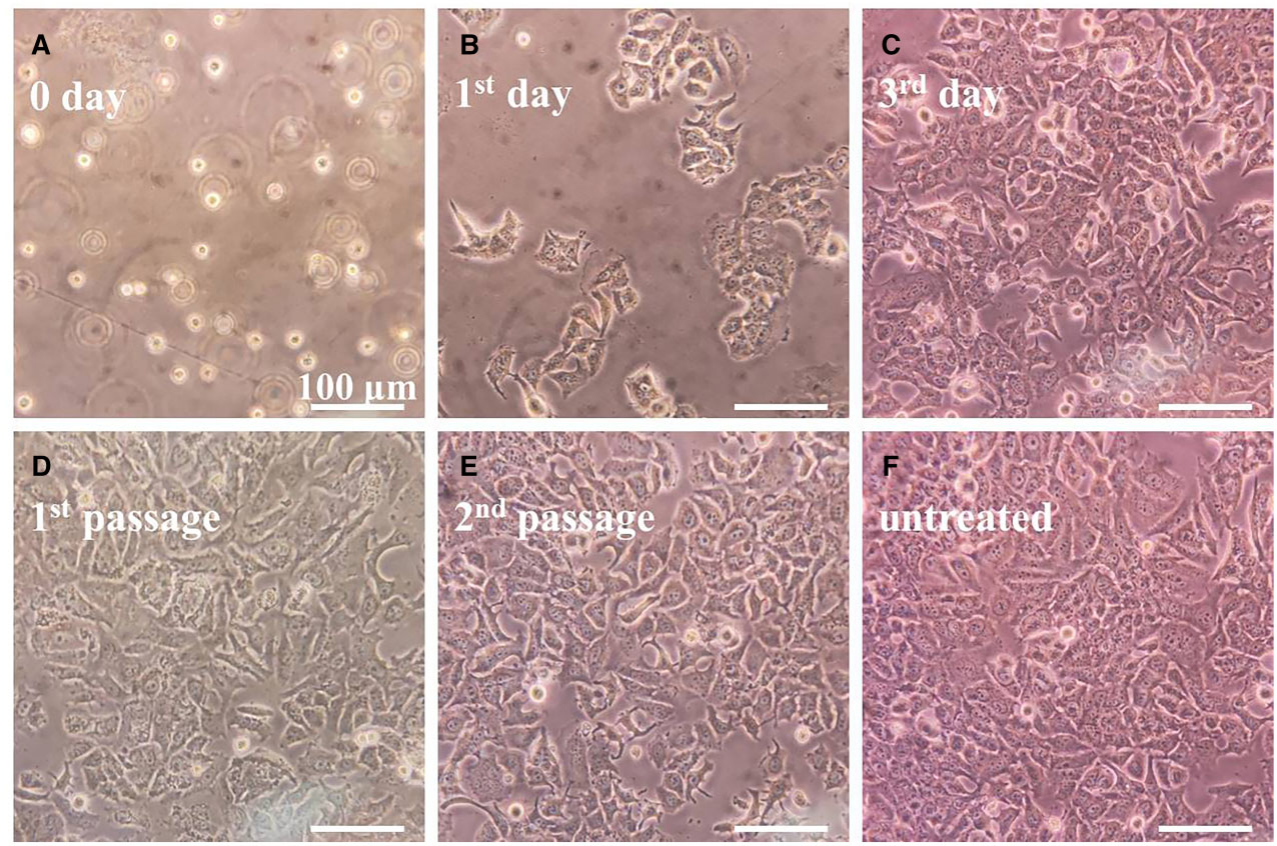
MCF-7 cells incubated with ASIMBs reculture in DMEM medium for (**A**–**C**) 0–3 days and after (**D**) first passage, (**E**) second passage; (**F**) showed the MCF-7 cells without incubating with ASIMBs.

In addition to the key step of CTCs’ enrichment, further downstream analyses, for example the proteomics, genomics and *ex vivo* culture are also important in the studies of CTCs, because these analyses would provide valuable biological information of the tumors and benefit clinic diagnosis and treatment [2–4]. On this basis, the captured MCF-7 cells were directly transferred to the Petri dishes and cultured at the same environment as untreated cells. Figure 9 shows that the cells could proliferate rapidly and colonize well to cover large areas of the Petri dishes within 3 days, and they could make a passage at least twice through the normal process. Compared to the untreated cells, the captured cells displayed no obvious changes in the morphology and the proliferation behavior (Supplementary Table S2, the mean area of captured MCF-7 cells was close to the untreated cells), which showed that these model CTCs enriched by our ASIMBs maintained good viability and could be used for the downstream analyses.

## Conclusions

We have successfully constructed a novel ASIMBs material, which integrated the advantages of the functional components (magnetic core, biocompatible PDA and GO layers, antifouling PEG layer and antibodies) through the facile LBL self-assembly strategy driven by π–π stacking. As-prepared ASIMBs could separate nearly 93% of MCF-7 cells, while <20% of Jurkat cells were nonspecifically captured, which revealed their excellent specificity and sensitivity to target cells. Besides, ASIMBs also showed remarkable capability to capture the rare MCF-7 cells from large amounts of surrounding Jurkat cells (capture efficiency: 75–100%) or mouse whole blood (capture efficiency: 20–90%). Meanwhile, the model CTCs separated by ASIMBs could be cultured directly without any other treatment, and no obvious changes in either cellular morphology or proliferation behavior were detected. All these results demonstrated that the ASIMBs hold promising capability to detect CTCs for potential clinical use, and the captured CTCs would be sufficiently qualified for the downstream analyses.

## Supplementary Material

rbad016_Supplementary_Data
