# Supplementary material for: Self-assembled supramolecular immunomagnetic nanoparticles through π–π stacking strategy for the enrichment of circulating tumor cells
Source: Regen Biomater. 2023 Mar 9;10:rbad016. doi: 10.1093/rb/rbad016 (PMC10070042; doi:10.1093/rb/rbad016)
Supplement: rbad016_Supplementary_Data [file rbad016_supplementary_data.docx]

**Supporting information**

**Self-assembled supramolecular immunomagnetic nanoparticles through π–π stacking strategy for circulating tumor cells enrichment**

Yanchao Mao^a^, Yujia Zhang^a^, Yue Yu^a^, Nanhang Zhu^a^, Xiaoxi Zhou^a^, Guohao Li^a^, Qiangying Yi﹡and Yao Wu﹡

**Author Information**

**Corresponding Authors**

Qiangying Yi − National Engineering Research Center for Biomaterials, Sichuan University, Chengdu 610064 Sichuan, P. R. China; orcid.org/0000-0002-9180-7322;

Email: [qyi@scu.edu.cn](mailto:qyi@scu.edu.cn)

Yao Wu − National Engineering Research Center for Biomaterials, Sichuan University, Chengdu 610064 Sichuan, P. R. China; orcid.org/0000-0002-0524-4042;

Email: [wuyao@scu.edu.cn](mailto:wuyao@scu.edu.cn)

**Authors**

a − National Engineering Research Center for Biomaterials, Sichuan University, Chengdu 610064 Sichuan, P. R. China


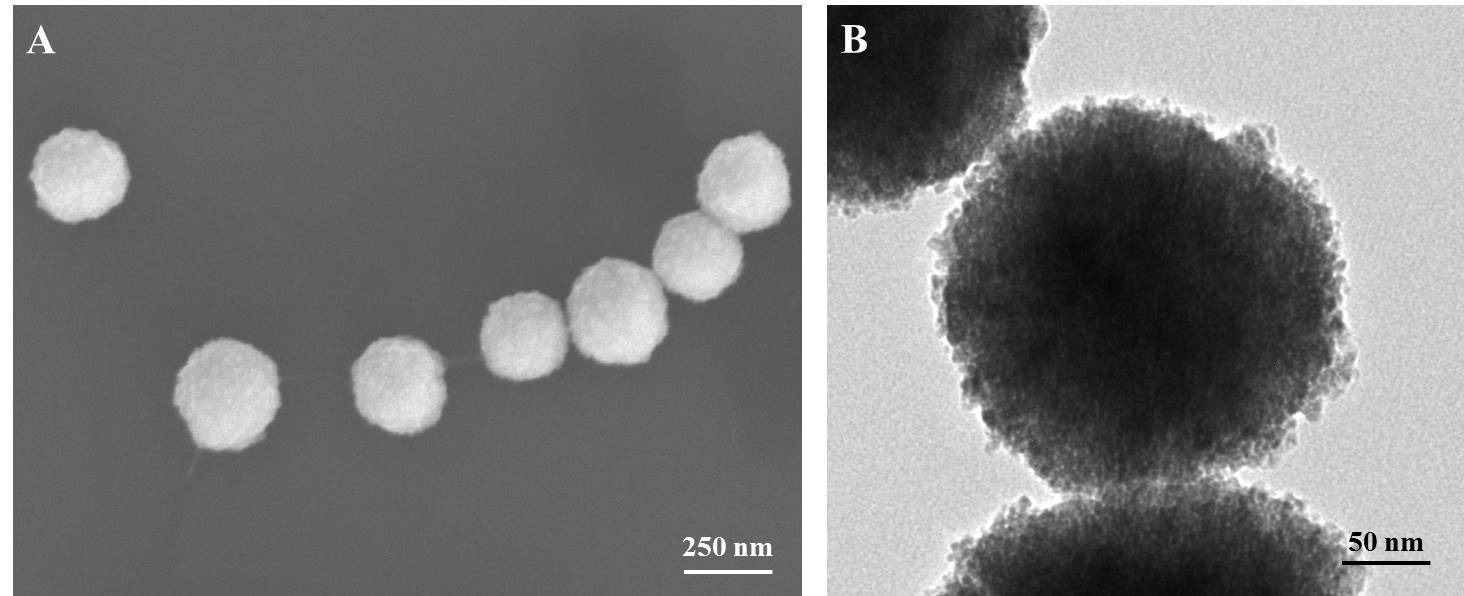


**Figure S1.** (A) SEM and (B) TEM photographs of MNPs.


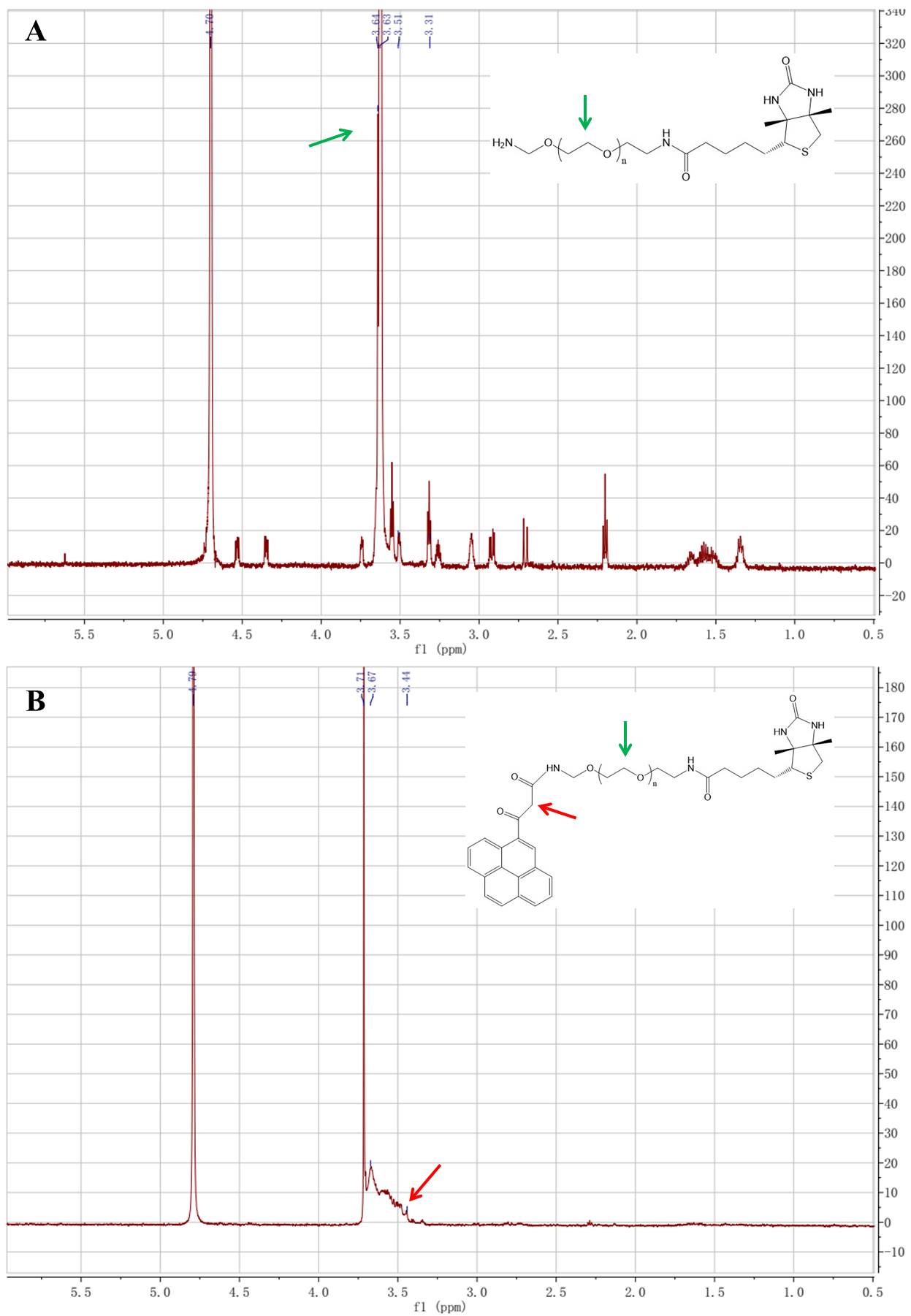


**Figure S2.** The ^1^H NMR spectra (600 MHz, D_2_O, room temperature) of (A) NH_2_-PEG-biotin and (B) OPBA-PEG-biotin. The chemical shifts at δ 3.44 (ppm) confirm the successful conjugation of the NH_2_-PEG-biotin and OPBA (red arrows), and the chemical shifts around δ 3.70 (ppm) represent the 4H in PEG (green arrows). The chemical shifts at δ 4.70 (ppm) represent the solvent.


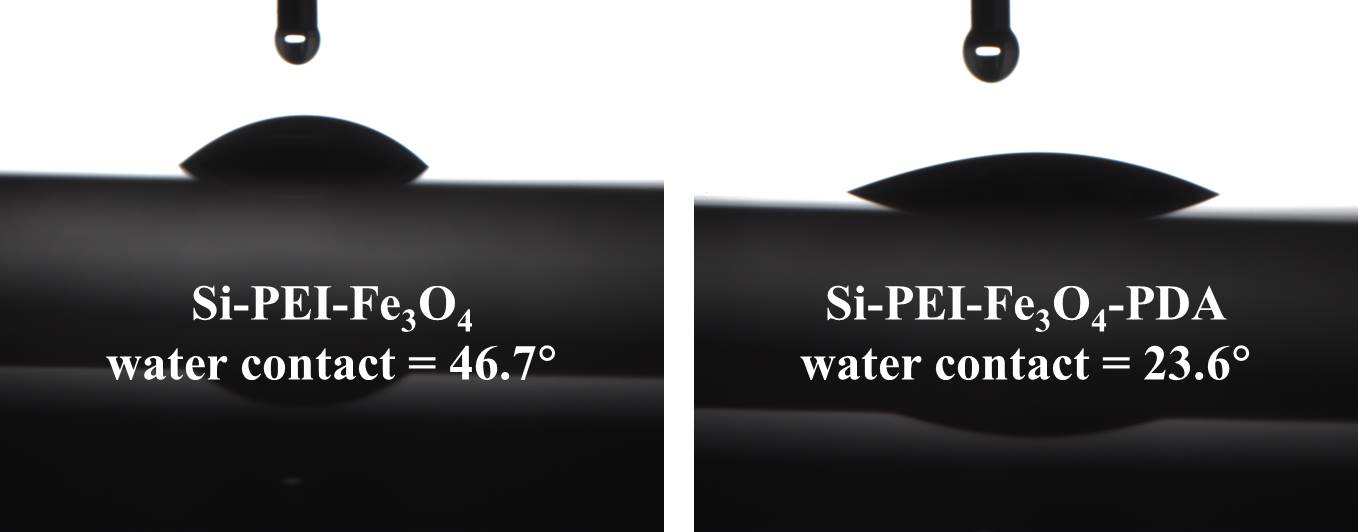


**Figure S3.** The water contact change after DA self-polymerization on the surface of PEI-Fe_3_O_4_.

**Table S1.** The comparison of ASIMBs and the similar methods for CTCs enrichment

| Construction of material | Capture efficiency for target cells | Capture efficiency for negative cells | References |
| --- | --- | --- | --- |
| Gold pattern@GO@PEG-linker-Ab | 87.3 % | Less than 10 % | 1 |
| MNP@QDs/PAH@HA-PEG-Ab | 92 % | 5 % | 2 |
| MNP@SiO_2_-Dendrimer-Ab | 85 % | 17 % | 3 |
| MNP@Au-MUA/MCH-Ab | Over 80 % | Less than 20 % | 4 |
| MNP@CM@Au-Ab | 79 % | 15 % | 5 |
| MACS® | ~70 % | ~27 % | 6 |
| Dynabeads® | ~80 % | ~10 % | 7 |
| MNP@PDA@GO@OPBA-PEG-Ab | 93 % | 17 % | This work |

**Table S2.** The mean area of attached cells in Figure 9

| Groups | Mean area of attached cells |
| --- | --- |
| 0 day | 18.52 |
| 1 day | 78.16 |
| 3 day | 144.57 |
| 1^st^ passage | 159.86 |
| 2^nd^ passage | 152.87 |
| untreated | 170.27 |

**References**

1. Yoon HJ, Kim TH, Zhang Z, Azizi E, Pham TM, Paoletti C, Lin J, Ramnath N, Wicha MS, Hayes DF, Simeone DM, Nagrath S. Sensitive capture of circulating tumour cells by functionalized graphene oxide nanosheets. Nat. Nanotechnol 2013; 8: 735-41.

2. Zhou X, Luo B, Kang K, Ma S, Sun X, Lan F, Yi Q, Wu Y. Multifunctional luminescent immuno-magnetic nanoparticles: toward fast, efficient, cell-friendly capture and recovery of circulating tumor cells. J Mater Chem B 2019; 7: 393-400.

3. Zhang PM, Zhang Y, Gao MX, Zhang XM. Dendrimer-assisted hydrophilic magnetic nanoparticles as sensitive substrates for rapid recognition and enhanced isolation of target tumor cells. Talanta 2016; 161: 925-31.

4. Gou YX, Liu JW, Sun CK, Wang P, You Z, Ren DH. Inertial-Assisted immunomagnetic bioplatform towards efficient enrichment of circulating tumor cells. Biosensors 2021; 11: 183-92.

5. Chang ZM, Zhou H, Yang C, Zhang R, You QN, Yan RH, Li L, Ge MF, Tang YG, Dong WF, Wang Z. Biomimetic immunomagnetic gold hybrid nanoparticles coupled with inductively coupled plasma mass spectrometry for the detection of circulating tumor cells. J. Mater. Chem. B 2020; 8:5019-25.

6. Zhou XX, Luo B, Kang K, Zhang YJ, Jiang PP, Lan F, Yi QY, Wu Y. Leukocyte-repelling biomimetic immunomagnetic nanoplatform for high-performance circulating tumor cells isolation. Small 2019; 15: 1900558-66.

7. Rao L, Meng QF, Huang QQ, Wang ZX, Yu GT, Li A, Ma WJ, Zhang NG, Guo SS, Zhao XZ, Liu K, Yuan YF, Liu W. Platelet–leukocyte hybrid membrane-coated immunomagnetic beads for highly efficient and highly specific isolation of circulating tumor cells. Adv. Funct. Mater. 2018; 28: 1803531-40.
